# Lectin nanoparticle assays for detecting breast cancer-associated glycovariants of cancer antigen 15-3 (CA15-3) in human plasma

**DOI:** 10.1371/journal.pone.0219480

**Published:** 2019-07-25

**Authors:** Joonas Terävä, Leena Tiainen, Urpo Lamminmäki, Pirkko-Liisa Kellokumpu-Lehtinen, Kim Pettersson, Kamlesh Gidwani

**Affiliations:** 1 Department of Biochemistry/Biotechnology, University of Turku, Turku, Finland; 2 Faculty of Medicine and Health Technology, Tampere University and Department of Oncology, Tampere University Hospital, Tampere, Finland; University of Liverpool, UNITED KINGDOM

## Abstract

Cancer antigen 15–3 (CA15-3) is widely utilized for monitoring metastatic breast cancer (BC). However, its utility for early detection of breast cancer is severely limited due to poor clinical sensitivity and specificity. The glycosylation of CA15-3 is known to be affected by BC, and therefore it might offer a way to construct CA15-3 glycovariant assays with improved cancer specificity. To this end, we performed lectin-based glycoprofiling of BC-associated CA15-3. CA15-3 expressed by a BC cell line was immobilized on microtitration wells using an anti-CA15-3 antibody. The glycosylation of the immobilized CA15-3 was then detected by using lectins coated onto europium (III)-doped nanoparticles (Eu^+3^-NPs) and measuring the time-resolved fluorescence of Eu. Out of multiple lectin-Eu^+3^-NP preparations, wheat germ agglutinin (WGA) and macrophage galactose-type lectin (MGL) -Eu^3+^-NPs bound to the BC cell line-dericed CA15-3 glycovariants (CA15-3^Lectin^). To evaluate the clinical performance of these two lectin-based assays, plasma samples from metastatic BC patients (n = 53) and healthy age-matched women (n = 20).Plasma CA15-3^Lectin^ measurements better distinguished metastatic BC patients from healthy controls than the conventional CA15-3 immunoassay. At 90% specificity, the clinical sensitivity of the assays was 66.0, 67.9 and 81.1% for the conventional CA15-3, CA15-3^MGL^ and CA15-3^WGA^ assays, respectively. Baseline CA15-3^MGL^ and CA15-3^WGA^ were correlated to conventional baseline CA15-3 levels (r = 0.68, p<0.001, r = 0.90, p>0.001, respectively). However, very low baseline CA15-3^MGL^ levels ≤ 5 U/mL were common in this metastatic breast cancer patient population.In conclusion, the new CA15-3^Lectin^ concept could considerably improve the clinical sensitivity of BC detection compared to the conventional CA15-3 immunoassays and should be validated further on a larger series of subjects with different cancer subtypes and stages.

## Introduction

Breast cancer (BC) is the most common cancer type and the second leading cause of cancer death in women worldwide [1]. Cancer antigen 15–3 (CA15-3 also known as MUC1) is shed from tumor cells and is a well-known serological marker for monitoring the clinical course of BC patients. A persistent increase in circulating concentration of this marker may suggest an inadequate response to cancer therapy in patients with metastatic BC. However, it has poor sensitivity, especially at early stages of the disease.[2] CA15-3 can also be elevated in healthy individuals and in patients with benign conditions, and it lacks the specificity needed for cancer screening, diagnosis, staging, and/or sole use in monitoring of post-therapy recurrence [3]. A study on retrospective samples found the sensitivity of the commercial Elecsys CA 15–3 immunoassay to be 7, 11, 39 and 78% on stage I, II, III and IV BC patients, respectively [4]. Recently an ultrasensitive, simple and reliable electrochemical immunosensor was developed to detect the lowest alteration of CA 15–3 and CA125, biomarker of breast and ovarian cancer patients respectively [5,6].

For monitoring metastatic breast cancer, international recommendations for the treatment of metastatic BC only recommend the monitoring of CA15-3 levels for patients with non-evaluable metastases, mainly bone-dominant disease [7,8]. Transient increases in plasma CA15-3 levels are possible without tumor progression [9]. This phenomenon is observed especially in the beginning of chemotherapy due to necrosis and apoptosis of tumor cells. Additionally, consensus about clinically meaningful increase in plasma CA15-3 levels to predict disease progression or clinically meaningful decrease to reflect a treatment response do not exist today. Nevertheless, in general plasma CA15-3 levels correlate with the response to chemotherapy in patients with metastatic breast cancer [10,11].

Protein glycosylation plays an important role in a wide variety of normal and disease-related biological processes. The phenomenon of aberrant glycosylation associated with malignant transformation, tumor progression and metastasis is well documented [12] and occurs in essentially all types of human cancers. A large number of altered glycosyl epitopes are classified as tumor-associated carbohydrate antigens. [13,14] Among these, the aberrant expression of Tn and sialyl-Tn antigens, L-fucose and terminal *N*-acetylglucosamine (GlcNAc) have been widely detected in breast cancers [15,16]. Especially, abnormal *O*-glycans, such as Tn antigen, are found in over 90% of breast cancers[17]. Overall, changes in glycosylation result in the production of various cancer-associated glycoproteins with cancer-associated glycoforms, which are antigenically distinct from the corresponding molecules of the normal tissue. Taking into account these modifications, the specificity of diagnostic cancer markers can be expected to be improved by using the aberrant glycoforms as targets.

CA15-3 is upregulated and aberrantly glycosylated in breast and other carcinomas [18].The CA15-3, derived from a large transmembrane protein Mucin 1 with molecular weight ranging from 500 to 1000 kDa, contains multiple O- and N-linked glycosylation sites. The *O*-glycans of CA15-3 produced by the normal breast tissue are core 2-based and can be complex, while the *O*-glycans added to the BC mucin are mainly core 1-based [19]. The resulting truncated glycans carried on BC-associated CA15-3 include Tn and T antigens and their sialylated forms [14]. CA15-3 purified from the culture medium of human BC YMB-S cells contains 3-O-sulfated or 3-sialylated core 1 and extended core 1 glycans. [20]

Glycans participate in early stages of tumorigenesis [12] and it has been reported that the expression level of an enzyme responsible for mucin-type glycosylation, N-acetylgalactosaminyltransferase-14, declines with breast cancer progression [21]. Thus, it is reasonable to assume that the cancerous glycovariants of glycoprotein tumor markers appear early and differ throughout the course of the disease. Therefore, glycovariant markers may be useful for early detection as well as for monitoring cancer progression.

Various lectins, members of a carbohydrate binding protein family, have previously been used to investigate the differences in glycosylation between soluble glycoproteins expressed by cancerous and benign tissues. A recent study described the use of a 3-sulfated core 1 -specific galectin-4 (Gal-4) to establish an assay exhibiting superior clinical performance compared to the conventional CA15-3 immunoassay for BC detection [22]. Also, C-type lectin receptors (CLR) such as macrophage galactose-type lectin (MGL) have been demonstrated to show increased binding to CA15-3 from lysates of colon cancer tissue compared to the healthy lysed colon tissues of the same patients [23]. The *Lens culinaris* agglutinin, a lectin found in lentil, in turn binds specifically to hepatocellular carcinoma -associated glycovariant of α-fetoprotein (AFP) and is the only lectin used in a commercial application to detect a biomarker glycovariant [24]. While showing these promising binding specificities, lectins unfortunately tend to have weak binding affinity, which apparently limits their exploitation in practical assay applications.

We previously reported a novel lectin-based approach for the detection of cancer-associated glycosylation of CA125, a well-known mucin 16 -derived cancer marker used e.g. for monitoring of epithelial ovarian cancer. The approach, relying on the use of highly fluorescent europium(III)-doped nanoparticles (Eu^+3^-NPs) coated with the lectin MGL, enabled highly sensitive detection of CA125 produced by ovarian cancer cell line OVCAR-3. In the clinical evaluation, the resulting optimized assay (CA125^MGL^) showed good discrimination between the samples of epithelial ovarian cancer patients and those with endometriosis, a condition that has decisively hampered the use of CA125 for early detection/screening of ovarian cancer. [25] In addition, we found that the new assay could alarm clinicians much earlier (4–6 months) than the conventional CA125 assay about disease relapse. These results motivated us to explore possibilities of the lectin nanoparticle assay concept for detecting the altered glycosylation of CA15-3 in the blood streams of BC patients.

In the present work, we utilized the lectin-Eu^+3^-NP approach for the glycoprofiling of CA15-3 with a panel of 28 lectins in order to identify lectins recognizing BC related changes in carbohydrate structures of CA15-3. The discovered promising lectins were then validated with plasma from patients with metastatic BC and healthy female controls. Additionally, we explored new CA15-3^lectin^ assays in monitoring response of metastatic breast cancer.

## Materials and methods

### Clinical samples

Plasma samples from 53 metastatic breast cancer patients were analyzed. These patients participated in a first-line chemotherapy trial for metastatic breast cancer (NCT00979641). The samples were analyzed at baseline, after six weeks of chemotherapy treatment, after six months of study treatment and at the final study visit. The trial design and the patient demographics have been published previously [26]. In brief, the patients with metastatic HER2-negative BC were enrolled into the trial, if they had not received previous chemotherapy for the advanced disease. The mean age of the study patients was 58 years (range 32–75). Most of the patients had hormone receptor positive disease (81%) and visceral metastases (79%). The median time between six-month sample and the final plasma sample was 11.8 months (inter quartile range 3.5.-18.9 months). The Ethics Committee of Tampere University Hospital approved the study protocol (R08142M). Clinically meaningful change in CA15-3 levels was defined as 30% similarly as the partial response criterion in the response evaluation criteria in solid tumors [27]. The definition of clinically meaningful change in circulating tumor markers varies around 20–40% in previous studies [10,28]. Disease progression was defined as investigator-assessed radiological progression according to the RECIST criteria [27].

Control plasma samples were obtained from 20 healthy women participating in a mammography-screening program in Tampere City Breast Clinic. These women voluntarily took part in a breast cancer primary prevention study currently in progress at University of Tampere and as a part of the study, plasma samples were drawn for scientific purposes. The mean age of these healthy controls was 56 years (range 54–67). All participants gave written informed consent (Ethics approval R15023).

### Reagents

CA15-3 isolated from the breast cancer cell line ZR-75-1 (ATCC CRL-1500) (BC-CA15-3), two monoclonal anti-CA15-3 antibodies; Ma552 and Ma695, that specifically recognize a PDTRPAPG region of the protein core and sialylated carbohydrate epitope expressed on the CA15-3 antigen respectively, were provided by Fujirebio Diagnostics (Göteborg, Sweden). Streptavidin-coated yellow 96-well plates, wash buffer and red assay buffer were purchased from Kaivogen (Turku, Finland). Europium(III)-doped Fluoro-Max polystyrene nanoparticles (97 nm in diameter) (Eu^+3^-NP) were acquired from Seradyn (Indianapolis, IN, USA). A panel of plant lectins with different glycan binding specificities (Table A in S1 Dataset) was obtained from Vector laboratories (Burlingame, CA, USA). The recombinant human lectins were purchased from R&D Systems (Abingdon, United Kingdom).

### Preparation of lectin-Eu^3+^-NPs

The use of Eu^+3^-NPs has been described before [29]. The coating of lectins on Eu^+3^-NPs was performed essentially as described before [30]. The buffer used for storage of the ^l^ectin coated Eu^+3^-NPs was 10 mM Tris-HCl, pH 7.8, supplemented with 0.1% BSA and 0.01% sodium azide at +4°C, covered from light. The particles were thoroughly vortexed and sonicated before every use to disperse aggregates.

### Labelling of antibodies with biotin

Both solid-phase monoclonal antibodies (Ma552 and Ma695 mAb) were biotinylated with 40-fold molar excess of biotin isothiocyanate, for 4 h at room temperature (RT). The labelled antibodies were separated from the unconjugated biotin by using NAP-5 and NAP-10 gel-filtration columns (GE Healthcare, Schenectady, NY, USA) by using 50 mM Tris–HCl (pH 7.75), containing 150 mM NaCl and 0.5 g/L NaN3. The labelled antibodies were stabilized with 1 g/L BSA (Bioreba, Nyon, Switzerland) and stored at +4°C. [31]

### In-house CA15-3 Lectin-NP assay

**Fig 1:**
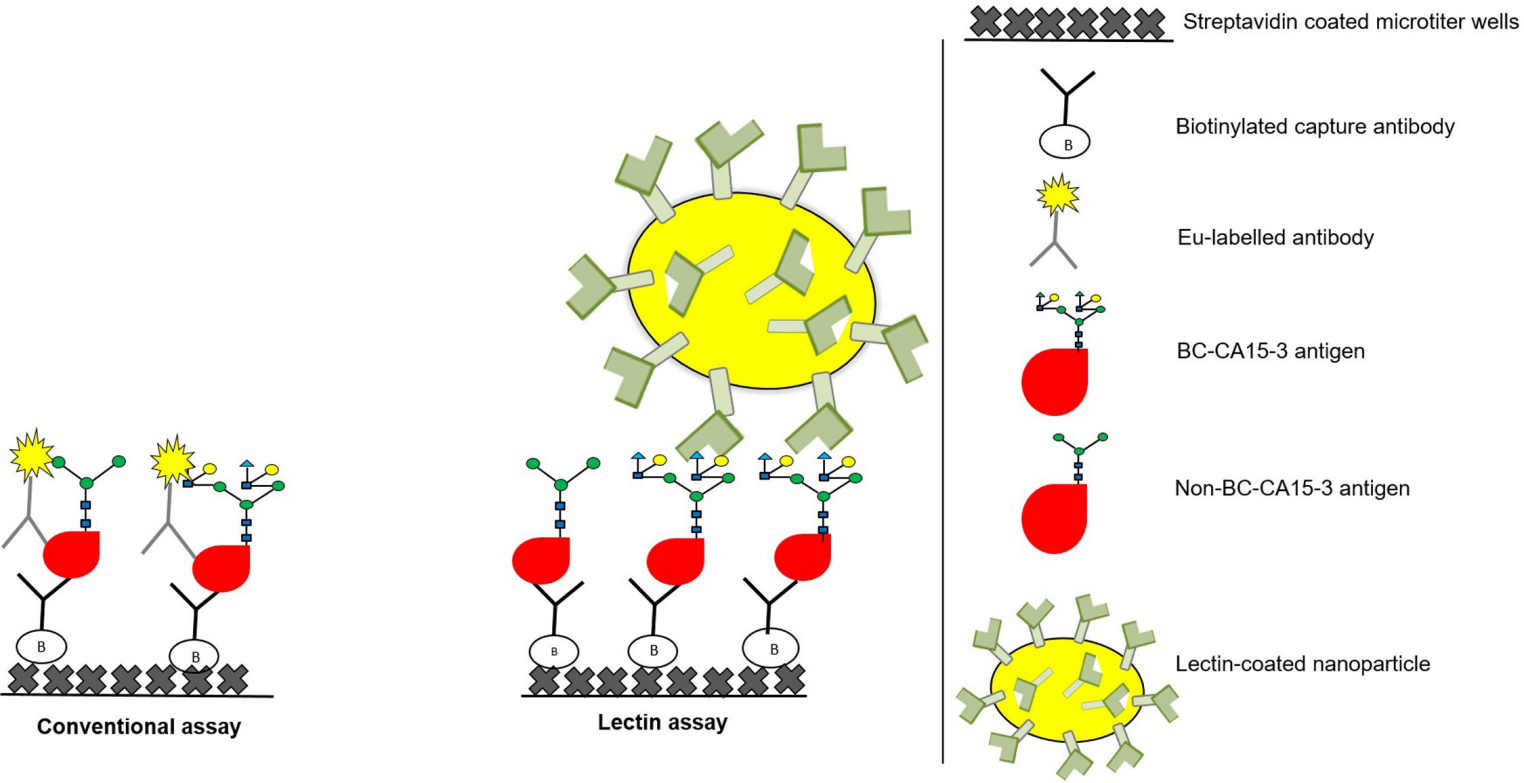
The principle of the conventional and in-house Eu^+3^-NP-based CA15-3 lectin assays. In the conventional CA15-3 immunoassay, the capture and tracer mAbs bind to the protein and glycan epitopes of CA15-3. Alternatively, in the lectin assay, the CA15-3 is captured with mAbs and detected with lectins, which have been coated on the surface of Eu^3+^-NPs. This method allows multivalent binding of the tracer to the glycan moieties of BC-CA15-3.

The assay principle is represented in Fig 1. Biotinylated Ma552 or Ma695 mAb (100 ng/30 μl/well) in buffer solution was incubated for 1 h at RT to immobilize them on streptavidin-coated yellow low-fluorescence microtiter wells. The wells were washed two times with wash buffer and 25 μl of CA15-3 standard/sample (diluted 1:40 in buffer) was added and incubated for 1 h at RT with slow shaking. The immobilized BC-CA15-3 was detected by lectin-Eu^3+^-NPs as a tracer by using time-resolved fluorescence (TRF). Ten million lectin Eu^+3^ -NPs per well in 25 μl of assay buffer containing additional 6 mM CaCl_2_ was added. The wells were incubated for two hours at RT in shaking and washed six times. To detect the lectin-Eu^3+^-NPs bound to BC-CA15-3, the TRF of Eu (λ_ex_: 340 nm; λ_em_: 615 nm) was measured for 400 μs after a 400 μs delay using Victor^3^V 1420 Multilabel counter (Wallac, Turku, Finland).

### Conventional CA15-3 immunoassay

CA15-3 concentrations were analysed in plasma samples with a CA15-3 enzyme immunoassay (Fujirebio Diagnostics Inc., Malvern, PA, USA) according to manufacturer’s instructions.

### Statistical analysis

Receiver operating characteristics (ROC) were determined and compared, and the areas under the curve (AUC) values calculated using R version 3.3. [32] with the pROC package [33]. The measured concentrations of each assay (Table B in S1 Dataset) were used as the classifier. The comparison of ROCs was done using the bootstrap method provided in the pROC package. Due to the nonparametric distribution of the CA15-3 levels, medians with the interquartile range (IQR) of the median were reported. CA15-3 levels of healthy controls were compared to CA15-3 levels of metastatic BC patients using the Mann Whitney *U*-test. Wilcoxon Rank test was used when comparing baseline and week six CA15-3 levels in relation to the treatment response. Spearman’s correlation was used to study the correlation between conventional CA15-3 levels and CA15-3^MGL^ or CA15-3^WGA^ levels. The Wilcoxon signed-rank test and Spearman’s correlation analyses were performed using SPSS version 23 statistical software package (SPSS Inc., Chicago, IL, USA). P value of less than 0.05 was considered significant in all statistical tests.

## Results

### Screening of lectins for binding to BC-CA15-3

**Fig 2:**
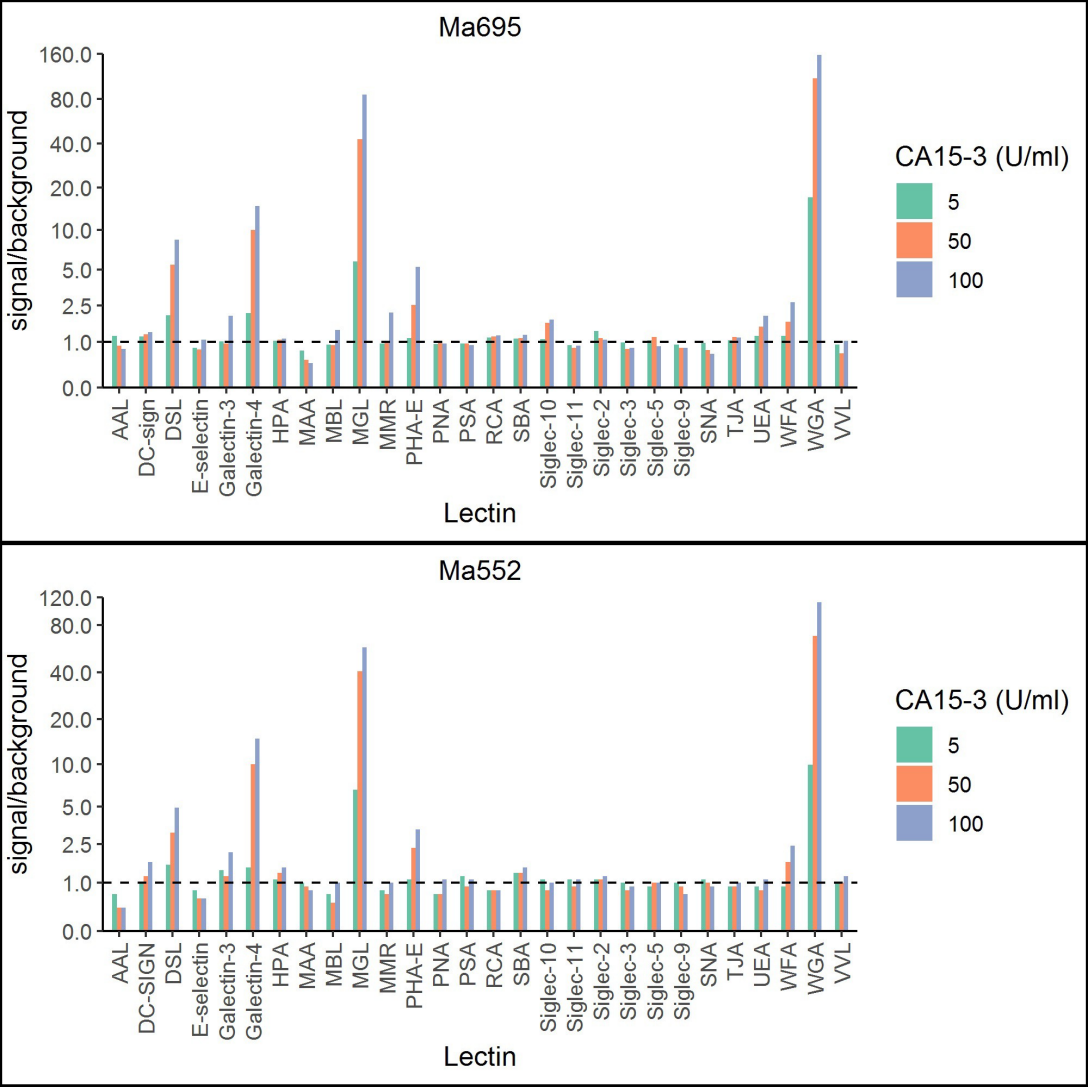
Lectin NPs binding to BC-associated CA15-3 from cell line ZR-75-1 (ATCC CRL-1500) using the lectin assay principle depicted in Fig 1. The different lectin Eu^+3^-NPs used are shown on the *x-axis* and the *y-axis* displays the signal to background ratios using either biotinylated Ma695 (bioMa695) or biotinylated Ma552 (bioMa552) as the capture mAb.

Altogether 28 lectins with various carbohydrate-binding specificities (Table A in S1 Dataset) were tested to investigate the glycosylation patterns of the cancer cell line -derived BC-CA15-3 preparation. Fig 2 shows the signal-to-background ratios obtained with the corresponding lectin-NP tracers using two different monoclonal antibodies (Ma552 and Ma695) for capturing CA15-3. Four of the tested nanoparticle tracers; MGL- WGA-, Gal-4-, and DSL-NPs, recognized BC-CA15-3 and the trend was similar for both capture antibodies. WGA exhibited highest signal-to-background ratio followed by MGL, Gal-4 and DSL (Fig 2). WGA- and MGL-NPs displayed excellent recovery (93% to 98%) when BC-CA15-3 was spiked into pooled healthy plasma samples whereas Gal-4- and DSL-NPs scarcely bound to BC-CA15-3 spiked similarly in plasma. We selected MGL (here after, CA15-3^MGL^) and WGA (CA15-3^WGA^) for further evaluation using clinical samples.

### Characteristics of CA15-3^MGL^ CA15-3^WGA^ assays

The analytical performance of the CA15-3^MGL^ and CA15-3^WGA^ assays were preliminarily tested using a BC-CA15-3 in a range of concentrations from 1 to 1000 U/mL. Saturation was not observed at the maximum used BC-CA15-3 of 1000 U/mL. The limit of detection, which was set to be the concentration of BC-CA15-3 required for a signal equivalent to the mean of blank calibrator (n = 20) plus three times the standard deviation, was less than 1 U/mL. Linear in response was observed at a maximum of 125 U/mL (S1 Fig). No cross-reactivity was observed towards two other glycoprotein cancer markers, CA125 and prostate specific antigen (S2 Fig).

### Plasma CA15-3, CA15-3^MGL^, and CA15-3^WGA^ concentrations in the study cohort

**Fig 3:**
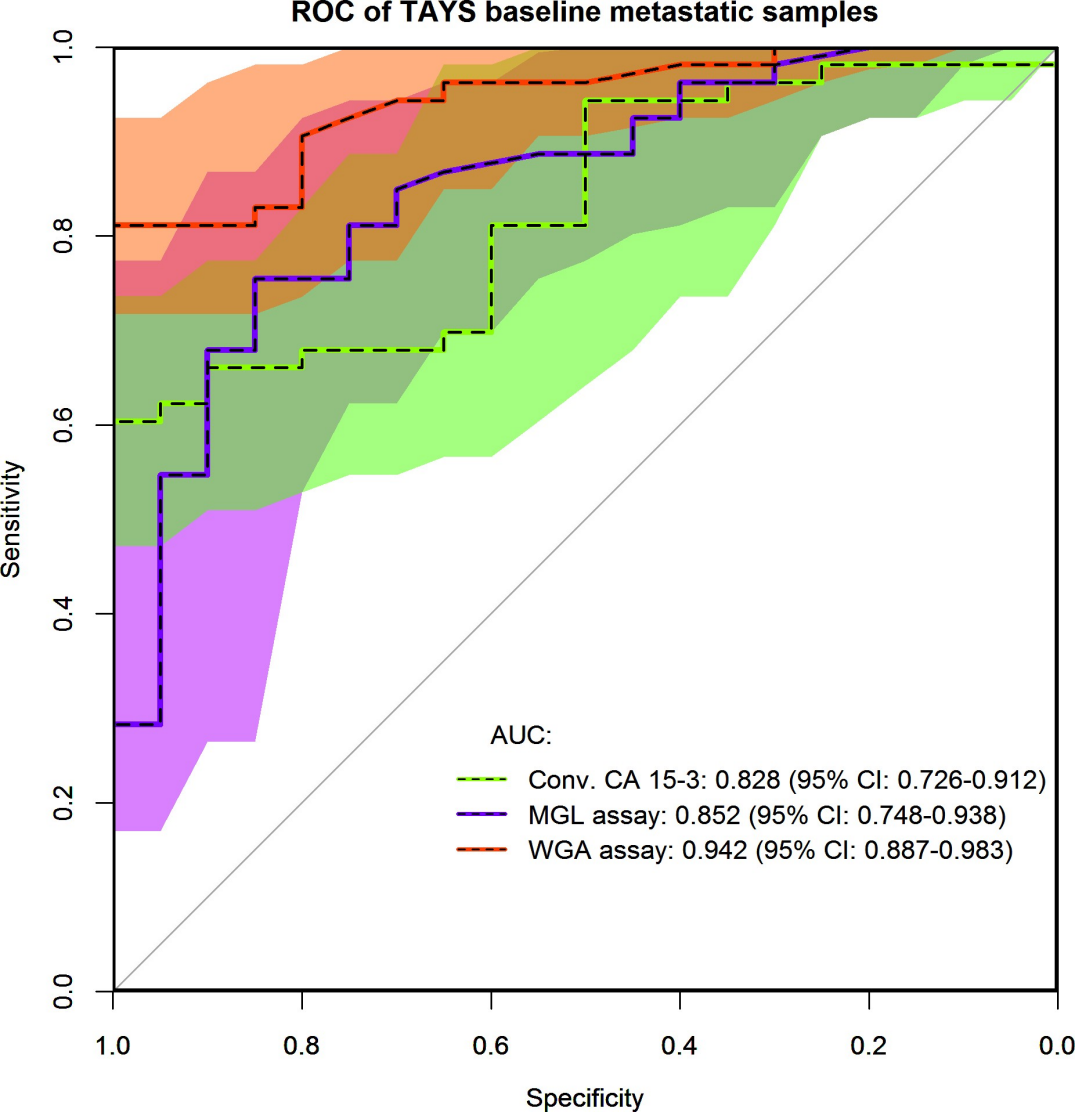
ROC plot displaying the AUC of conventional CA15-3 (green), CA15-3^MGL^ (purple) and CA15-3^WGA^ (red) from metastatic breast cancer patients (n = 53) and healthy control (n = 20). The 95% confidence intervals of the ROCs are depicted as shaded areas and displayed numerically in brackets. The color of shadings corresponds to the plotted lines and the overlap of conventional CA15-3 and CA15-3^MGL^ is dark green, the overlap of all assays is brown and the overlap of CA15-3^MGL^ and CA15-3^WGA^ is dark red.

We next studied whether CA15-3 in the plasma of BC patients binds with MGL and WGA similar to CA15-3 of a breast cancer cell line. The baseline EDTA plasma samples from 53 patients with metastatic BC and 20 healthy individuals were measured for CA15-3^MGL^ and CA15-3^WGA^ and compared with the conventional CA15-3 immunoassay. To assess the diagnostic value of the tumor markers in metastatic BC, ROC curves were plotted and AUC was calculated. The highest AUC value was achieved with CA15-3^WGA^ (0.943) followed by CA15-3^MGL^ (0.852) while the conventional CA15-3 immunoassay yielded the lowest AUC of 0.827 (Fig 3). At 90% specificity the sensitivities of the assays were 81.1, 67.9 and 66.0% for the CA15-3 ^WGA^, CA15-3^MGL^ and conventional CA15-3, respectively. The difference in the AUC compared to the conventional assay was significant for CA15-3^WGA^ (p = 0.007) but not for CA15-3^MGL^ (p = 0.655).

Metastatic BC patients had higher median baseline plasma levels of conventional CA15-3 as well as CA15-3^MGL^ and CA15-3^WGA^ levels than the healthy controls (Table 1). Plasma samples were available from 53 metastatic breast cancer patients. However, both baseline and week six samples were available only from 41 patients. Median CA15-3 levels were lower at week six than at baseline for all three CA15-3 assays in the entire study population (p-values 0.007, <0.001, <0.001 for CA15-3, CA15-3^MGL^ and CA15-3^WGA^, respectively). The decline in CA15-3 levels was more pronounced in responding patients for all CA15-3 assays, especially CA15-3^MGL^ (Table 2). For all the three different CA15-3 assays, the responding patients had a significant decrease in all assays of CA15-3 between baseline and week six (p-values 0.003, <0.001 and <0.001 for CA 15–3, CA15-3^MGL^ and CA15-3^WGA^, respectively, Table 2).

**Table 1:** CA15-3 levels by conventional CA15-3, CA15-3^MGL^, and CA15-3^WGA^ assay for healthy controls and for metastatic BC patients at study baseline.

	n	Conventional CA15-3	CA15-3^MGL^	CA15-3^WGA^
**Healthy controls**				
Median CA15-3 U/mL (IQR)	20	13.3 (7.9–23.1)	2.0 (0.2–3.6)	1.6 (0.5–2.7)
**Metastastic BC patients**, Baseline				
Median CA15-3 U/mL (IQR)	53	47.4 (18.9–99.9)	4.4 (1.3–16.5)	7.0 (3.1–41.0)
p-value^a^		<0.001	0.013	<0.001

Abbreviations: n = number of patients, IQR = interquartile range, BC = breast cancer

^a^ Mann-Whitney *U*-test

**Table 2:** CA15-3 levels with different assays depending on the best response to the chemotherapy treatment.

	n	Baseline Median CA15-3, U/mL (IQR)	Week 6 Median CA15-3 U/mL (IQR)	Change, median % (IQR)^a^	p^b^	Declining CA15-3 levels, n (%)^c^	Increase in CA15-3 levels, n (%)^d^
Conventional CA15-3							
PR	25	71.1 (29.4–228)	55.4 (28.8–103)	-23.8 [-52.7-(-14.0)]	**0.003**	10 (40.0)	2 (8.0)
SD	14	19.2 (12.6–81.4)	25.8 (14.9–71.0)	-0.4 (-37.1–60.5)	0.875	4 (28.6)	5 (35.7)
PD	2	24.1 (15.0–33.1)	29.2 (17.5–40.9)	+20.1 (16.7–23.6)	0.180	0	0
CA15-3^MGL^							
PR	25	6.3 (2.1–45.1)	2.4 (0.9–4.8)	-75.0 [-86.4-(-41.0)]	**<0.001**	18 (78.2)	3 (13.0)
SD	14	3.2 (1.0–5.4)	2.2 (0.8–3.6)	-33.3 (-67.0–33.3)	**0.036**	7 (53.8)	4 (30.7)
PD	2	4.1 (3.2–5.0)	3.0 (2.2–3.9)	-17.0 (-56.0–21.9)	0.655	1 (50.0)	0
CA15-3^WGA^							
PR	25	13.2 (5.3–76.5)	8.0 (3.5–33.2)	-27.2 [-55.9-(-19.4)]	**<0.001**	12 (48.0)	2 (8.0)
SD	14	3.2 (2.4–8.7)	5.0 (2.7–8.2)	+22.2 (-25.4–60.0)	0.851	3 (21.4)	7 (50.0)
PD	2	4.7 (2.3–7.0)	5.8 (2.3–9.3)	+16.4 (0–32.9)	0.317	0	1 (50.0)

Abbreviations: n = number of patients, CI = confidence interval, PR = partial response, SD = stable disease, PD = progressive disease

^a^ Change in CA15-3 levels from baseline to week six in percentiles, median

^b^ Wilcoxon Rank Test

c Patients with ≥ 30% decline in CA15-3 levels from baseline to week six

d Patients with ≥ 30% increase in CA15-3 levels from baseline to week six

**Fig 4:**
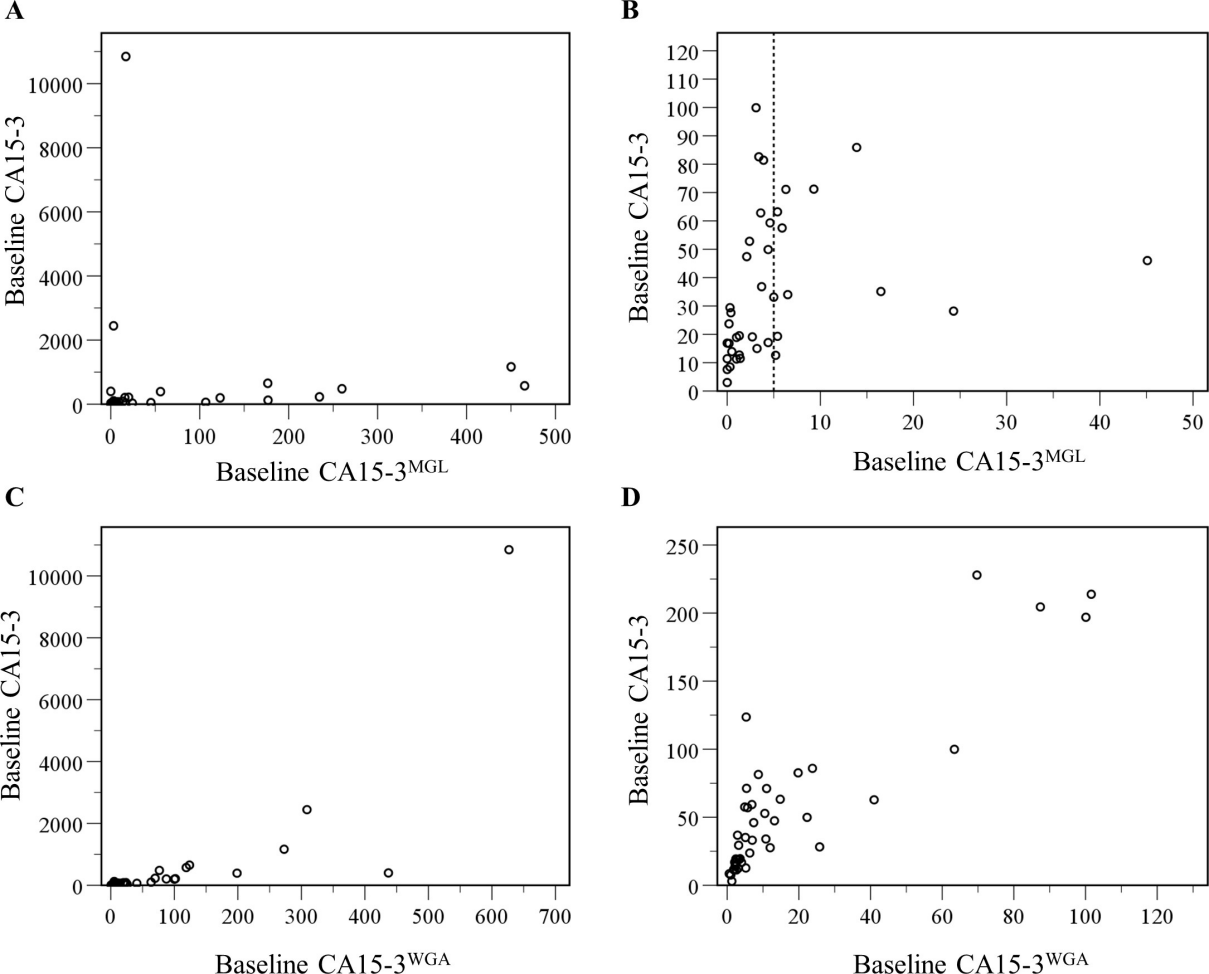
Correlation of the conventional CA15-3 and CA15-3^Lectin^ assays. **A:** Scatterplot of baseline conventional CA15-3 levels and baseline CA15-3^MGL^ levels in metastatic breast cancer patients. r = 0.68, p<0.001 **B:** Enlargement of the scatterplot for the patients with the lowest CA15-3 levels for both conventional CA15-3 and CA15-3^MGL^. Very low baseline CA15-3MGL levels < 5 U/mL were observed in 29 patients (44.6%), dashed vertical line at x-axis **C:** Scatterplot of baseline conventional CA15-3 levels and baseline CA15-3^WGA^ levels in metastatic breast cancer patients. r = 0.90, p<0.001. **D:** Enlargement of the scatterplot for the patients with conventional CA15-3 < 250 U/mL and CA15-3WGA < 130 U/mL, 85% of the study patients.

Baseline conventional CA15-3 and CA15-3^MGL^ levels correlated to each other (r = 0.68, p<0.001, Fig 4A and 4B). However, almost half of the metastatic BC patients had very low baseline CA15-3^MGL^ levels (≤ 5 U/Ml, dashed vertical line in Fig 4B). A stronger correlation was observed between conventional CA15-3 and CA15-3^WGA^ (r = 0.90, p<0.001, Fig 4C and 4D).

**Fig 5:**
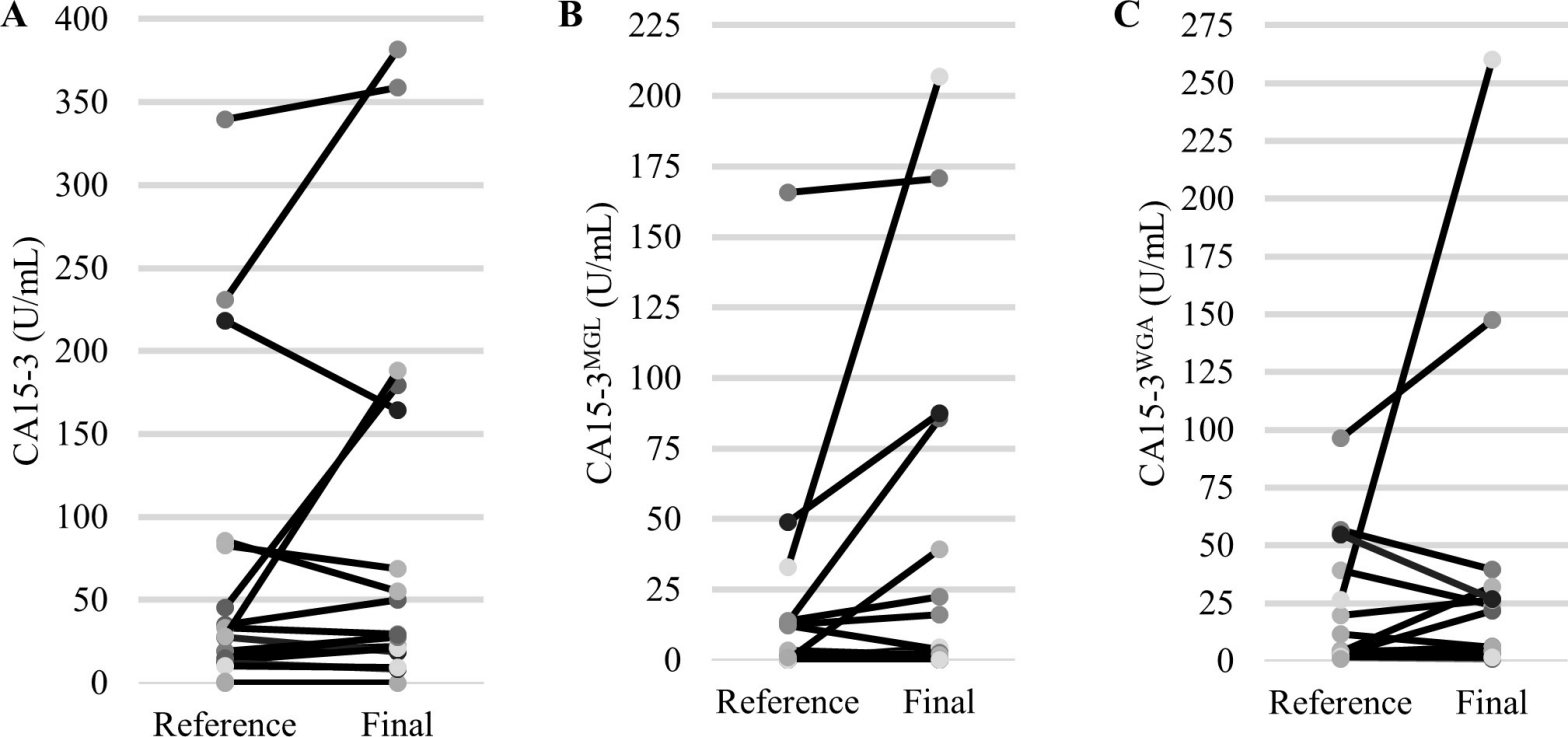
CA15-3 levels of 19 patients who had a disease progression at the time of final plasma sampling and a previous reference plasma available while on study treatment (Reference). **A.** Conventional CA15-3. Two patients with very high CA15-3 levels were excluded (Reference CA15-3 110.6 U/mL, Final 986.4 U/mL and Reference 1825.5 U/mL, Final 3909.7 U/mL) **B.** CA15-3^MGL^. **C.** CA15-3^WGA^. One patient with very high CA15-3^WGA^ level was excluded (Reference CA15-3^WGA^ 393.1 U/mL, Final CA15-3^WGA^ 430.9 U/mL).

Additionally, we studied CA15-3 levels at disease progression (Fig 5).We had plasma samples available from 19 patients who had a disease progression at final study visit. A clinically meaningful 30% increase in the final CA15-3 levels was observed in eight patients (42%) with the conventional CA15-3, nine patients (47%) with the CA15-3^MGL^ and six patients (32%) with the CA15-3^WGA^. The patients with rising CA15-3 levels at disease progression were not entirely the same individuals for the different CA15-3 assays. Specifically, five patients had similar increase in final CA15-3^MGL^ levels and CA15-3 levels. However, four patients with rising CA15-3^MGL^ levels did not have an increase in conventional CA15-3 levels. Furthermore, a similar increase was observed in four patients in final CA15-3^WGA^ and conventional CA15-3 levels. However, two patients with rising CA15-3^WGA^ levels did not have an increase in conventional CA15-3 levels. Additionally, at least 30% decrease in the final CA15-3 levels at disease progression was observed in three patients (16%) with the conventional CA15-3, 3 patients (16%) with CA15-3^MGL^ and five patients (26%) with CA15-3^WGA^.

## Discussion

The results suggest that the glycovariant specific assays provide advantages over the conventional CA15-3 immunoassay in monitoring of BC patients, and especially for the detection of metastatic disease and its recurrence.

CA15-3 is a tumor marker commonly used for monitoring patients with advanced BC. However, the currently employed sandwich immunoassays that target two protein epitopes have moderate clinical sensitivity and specificity. [34] While it has been established that abnormal glycosylation occurs in cancers and there has been investigations into multiple different approaches for their detection [35] the efforts to further develop the CA15-3 based diagnostic assay have been limited. The changes in glycosylation can lead to altered interactions of glycoproteins expressed by the tumor cell with different lectins. The development of glycoprofiling assays for blood-derived products has been made difficult by the fact that cancer specific glycovariants may only exist in small amounts in blood and are therefore problematic to detect. We have previously utilized the lectin-NP -based platform successfully to explore the glycosylation of serum glycoprotein CA125 in ovarian cancer patients [25]. Two features in a combination enhance the analytical sensitivity of lectin-NPs; 1) signal amplification provided by the thousands of Eu chelates doped in a single particle and 2) the strengthening of the functional affinity (avidity) of the lectins to their target glycostructure epitopes enabled by the high-density immobilization of lectin on the particles.

The present study shows for the first time that a qualitative glycovariant assay to detect the changes of the CA15-3 glycoprotein can improve on the diagnostic utility of current assays. We developed an assay for sensitive and quantitative detection of aberrant glycosylation on BC-CA15-3 providing enhanced preference for the cancer-associated glycovariant of the tumor marker. An antibody recognizing the protein/glycan epitopes of CA15-3 was used for the immobilization and a panel of lectins was tested for the ability to bind the immobilized CA15-3. The tested panel of 28 lectins covers a variety of common human glycans. Only two of the tested lectin-Eu^+3^-NP preparations exhibited satisfactory binding to the BC-associated CA15-3. Those lectins were WGA and recombinant human MGL. WGA and MGL recognize the GlcNAc and GalNAc -containing epitopes respectively, frequently expressed on the surface of cancer cells [36–38]. Using CA15-3^WGA^ and CA15-3^MGL^ assays, in the plasma of metastatic BC patients are likely to serve as more cancer-specific than the conventional assay. In patients with metastatic BC, the newly developed CA15-3^WGA^ assay was able to detect 81% compared to 66% with conventional CA15-3 assay when only 10% of controls were misdiagnosed with both assays.

Consistent with these findings, Nollau P *et al*. describe the use of recombinant MGL (also known as CLEC10A), for the detection of ligands in sections from formalin-fixed, paraffin-embedded normal and cancerous mammary tissues. In comparison to normal mammary glands, a pronounced staining of tumour tissues was observed. [37] Beatson R *et al*. also observed that MUC1 carrying both Tn and STn epitopes can bind to the human lectin MGL, and using atomic force microscopy they showed that Tn and sTn bind to MGL with a similar de-adhesion force. [39] Our study reports the binding of that same human lectin MGL with plasma of BC patients and particularly with CA15-3. Blixt *et al*. reported that high levels of a subset of autoantibodies to the core 3-MUC1 (GlcNAcβ1-3GalNAc-MUC1) and STn-MUC1 glycoforms were significantly associated with reduced incidence and increased time to metastasis, which also supports our findings of MGL binding [18].

As far as we know, this study is the first to report WGA’s specificity for BC-CA15-3. WGA is a plant lectin, which specifically recognizes the sugars NeuNAc and GlcNAc [39]. It has been reported that terminal GlcNAc is characteristic of a group of protein- and lipid-linked glycans overexpressed in many malignant tumor tissues including breast carcinoma [16]. Chandrasekaran EV *et al* studied the complex carbohydrate-lectin interactions by determining the effects of substituents in mucin core 2 tetrasaccharide Galβ1-4GlcNAcβ1-6(Galβ1–3) GalNAcα-OR and fetuin glycopeptides on their binding to agarose immobilized lectin WGA. Compounds with α2-3-sialyl T-hapten, α2-6-Sialyl LacdiNAc, α2-3-sialyl D-Fucβ1–3 GalNAc and Fucα-1-2 D-Fucβ-1-3GalNAc displayed regular binding and GalNAc, LewisX and LacdiNAc plus D-Fuc β-1-3 GalNAcα exhibited particularly tight binding.[40] A previous study by Bird-Liebermann EL *et al* identified GlcNAc as a biomarker for endoscopic visualization of Barrett’s esophagus to detect dysplastic esophageal tissue [41]. Using a serum CA15-3 lectin assay based on antibody-capture, Ideo *et al*. showed that 3-sulfated core 1 specific Gal-4 can be used to measure CA15-3 that is present in BC[22]. We observed in our study that Gal-4 bound more poorly to BC-CA15-3 than MGL and WGA.

The specificity provided by the immobilizing antibody together with the glycan-recognition of the lectins, which is enhanced through the avidity made possible by the Eu+3-NPs, constitute the technical concept behind the novel CA15-3^MGL^ and CA15-3^WGA^ assays. These assay strongly prefer the cancer-associated glycovariants compared to conventional CA15-3 sandwich immunoassay. Based on previously published research on the nature of CA15-3 glycosylation in malignant and benign states, the WGA preference for cancer CA15-3 would have been difficult to predict. It is possible that the glycan eiptopes reactive with MGL and WGA are be present on several CA15-3 glycans, being a sizable 500–1000 kDa glycoprotein. The extracellular domain of CA15-3 consists mainly of 25–150 tandem repeats of 20 amino acids. Each repeat carries five *O*-linked glycosylation sites, thus glycans can potentially be repeated 125–750 times on each molecule allowing engagement of relevant lectins.[20] The high amount of glycans makes the presence of multiple binding sites for MGL and WGA Eu^+3^-NPs plausible and may provide for high avidity even at low CA15-3 concentration. The low limit of detection and great linearity of the standard in the range of 1-100 U/ml of analyte agrees with this assessment.

The monitoring of conventional CA15-3 levels for therapy response of metastatic BC is currently recommended only as an adjunctive assessment to aid clinical decisions[42].This is mostly due to low sensitivity and specificity of the conventional CA15-3 assay. Additionally, the conventional CA15-3 levels may have discrepancies compared to clinical findings and radiological assessments[10]. Although for majority of patients, the tumor markers decline in responding patients and increase in progressing patients, misinterpretations are possible if tumor markers are evaluated alone for an individual patient. In our study, 10 patients (40%) with a partial response to study treatment had at least 30% decline in conventional CA15-3 levels between baseline and week six. However, declining plasma levels for responding patients were more common both with the CA15-3^MGL^ method (18 patients, 78%) and the CA15-3^WGA^ method (12 patients, 48%). Therefore, new CA15-3^lectin^ assays recognize the responding patients better than the conventional CA15-3. False positive increases in CA15-3 levels were observed for responding patients for all three assays between baseline and week six (two patients for conventional CA15-3 and CA15-3^WGA^, three patients for CA15-3^MGL^). At the time of progressive disease, only minority of patients had over 30% increase in their CA15-3 levels [conventional CA15-3 assay 8 patients (42%), CA15-3^MGL^ 9 patients (47%), CA15-3^WGA^ 5 patients (26%)].

Comparing the new lectin assays to one another, CA15-3^WGA^ seems to be more suitable for clinical use than CA15-3^MGL^. The clinical utility for CA15-3^MGL^ levels is limited due to very low < 5 IU/mL baseline levels detected for almost half of our study patients. Additionally, the correlation between conventional CA15-3 and CA15-3^WGA^ was more pronounced (r = 0.90, p<0.001). Nevertheless, for this limited patient population, CA15-3^MGL^ and CA15-3^WGA^ seem not yet to be ideal assays for clinical utility and the possibilities for misinterpretations for an individual patient remains as it does for the conventional CA15-3 assay. However, it would be worthwhile test these CA15-3^lectin^ assays in a prospective trial involving metastatic breast cancer patients.

This study suggests that using CA15-3 mAb and WGA and MGL Eu3+NPs are more sensitive in distinguishing metastatic BC patients from healthy controls than conventional CA15-3 immunoassay.

Due to the limited amount of patient samples used in this proof of concept study report, studies for further validation, to establish the clinical performance of CA15-3^WGA^ and CA15-3^MGL^ assays for BC surveillance, and monitoring progression and therapeutic responses of metastatic disease, are now under investigation. The findings also warrant further investigation of this approach in other cancers.

## Supporting information

S1 Fig **Calibration curves of (A) CA15-3 WGA and (B) CA15-3-MGL NPs-lectin assay.** Both are linear in range of 1 to 125 U/ml with excellent analytical sensitivity.
(PPTX)

S2 Fig Assay cross-reactivity with other common tumor markers. No cross reactivity with CA15-3 lectin assay was observed with ovarian cancer cell line associated CA125 and prostate cancer associated LnCAp PSA
(PPTX)

S1 Dataset Table A. Lectins used. Table B. Concentrations measured from controls and metastatic cases (baseline samples).
(DOCX)

S2 Dataset Patient cohort data. (SAV)

## References

[1] Cancer society of Finland. Statistics and research—Syöpärekisteri [Internet]. 2018 [cited 10 Oct 2018]. Available: https://cancerregistry.fi/

[2] Al-Azawi D, Kelly G, Myers E, McDermott EW, Hill ADK, Duffy MJ, et al CA 15–3 is predictive of response and disease recurrence following treatment in locally advanced breast cancer. BMC Cancer. 2006;6: 3–9. 10.1186/1471-2407-6-3 16953875 PMC1590047

[3] Sturgeon CM, Hoffman BR, Chan DW, Ch’ng S-L, Hammond E, Hayes DF, et al National Academy of Clinical Biochemistry Laboratory Medicine Practice Guidelines for Use of Tumor Markers in Clinical Practice: Quality Requirements. Clin Chem. 2008;54: e1–e10. 10.1373/clinchem.2007.094144 18606634 PMC5564312

[4] Stieber P, Molina R, Chan DW, Fritsche HA, Beyrau R, Bonfrer JMG, et al Clinical evaluation of the elecsys CA 15–3 test in breast cancer patients. Clin Lab. 2003;49: 15–24. Available: http://www.ncbi.nlm.nih.gov/pubmed/12593471 12593471

[5] Akbari Nakhjavani S, Khalilzadeh B, Samadi Pakchin P, Saber R, Ghahremani MH, Omidi Y. A highly sensitive and reliable detection of CA15-3 in patient plasma with electrochemical biosensor labeled with magnetic beads. Biosens Bioelectron. Elsevier B.V.; 2018;122: 8–15. 10.1016/j.bios.2018.08.047 30236808

[6] Samadi Pakchin P, Ghanbari H, Saber R, Omidi Y. Electrochemical immunosensor based on chitosan-gold nanoparticle/carbon nanotube as a platform and lactate oxidase as a label for detection of CA125 oncomarker. Biosens Bioelectron. Elsevier B.V.; 2018;122: 68–74. 10.1016/j.bios.2018.09.016 30243046

[7] NCNN. Clinical Practice guidelines in oncology [Internet]. 2018 [cited 21 Sep 2018]. Available: http://www.nccn.org

[8] Cardoso F, Senkus E, Costa A, Papadopoulos E, Aapro M, André F, et al 4th ESO–ESMO International Consensus Guidelines for Advanced Breast Cancer (ABC 4)†. Ann Oncol. 2018;29: 1634–1657. 10.1093/annonc/mdy192 30032243 PMC7360146

[9] Kim HS, Park YH, Park MJ, Chang MH, Jun HJ, Kim KH, et al Clinical significance of a serum CA15-3 surge and the usefulness of CA15-3 kinetics in monitoring chemotherapy response in patients with metastatic breast cancer. Breast Cancer Res Treat. 2009;118: 89–97. 10.1007/s10549-009-0377-2 19322651

[10] Tampellini M, Berruti A, Bitossi R, Gorzegno G, Alabiso I, Bottini A, et al Prognostic significance of changes in CA 15–3 serum levels during chemotherapy in metastatic breast cancer patients. Breast Cancer Res Treat. 2006;98: 241–248. 10.1007/s10549-005-9155-y 16670941

[11] Kurebayashi J, Nishimura R, Tanaka K, Kohno N, Kurosumi M, Moriya T, et al Significance of serum tumor markers in monitoring advanced breast cancer patients treated with systemic therapy: a prospective study. Breast Cancer. 2004;11: 389–395. 10.1007/BF02968047 15604995

[12] Fuster MM, Esko JD. The sweet and sour of cancer: Glycans as novel therapeutic targets. Nat Rev Cancer. Nature Publishing Group; 2005;5: 526–542. 10.1038/nrc1649 16069816

[13] Hakomori SI. Tumor malignancy defined by aberrant glycosylation and sphingo(glyco)lipid metabolism. Cancer Res. American Association for Cancer Research; 1996;56: 5309–5318. Available: http://www.ncbi.nlm.nih.gov/pubmed/8968075 8968075

[14] Heliström I, Raycraft J, Hayden-Ledbetter M, Ledbetter JA, Schummer M, McIntosh M, et al The HE4 (WFDC2) protein is a biomarker for ovarian carcinoma. Cancer Res. 2003;63: 3695–3700. 10.1073/PNAS.172380699 12839961

[15] Brooks SA, Leathem AJM. Expression of alpha-GalNAc glycoproteins by breast cancers. Br J Cancer. 1995;71: 1033–1038. 10.1038/bjc.1995.199 7537516 PMC2033780

[16] Satomaa T, Heiskanen A, Leonardsson I, Ångström J, Olonen A, Blomqvist M, et al Analysis of the human cancer glycome identifies a novel group of tumor-associated N-acetylglucosamine glycan antigens. Cancer Res. 2009;69: 5811–5819. 10.1158/0008-5472.CAN-08-0289 19584298

[17] Song K, Herzog BH, Fu J, Sheng M, Bergstrom K, McDaniel JM, et al Loss of core 1-derived O-glycans decreases breast cancer development in mice. J Biol Chem. 2015;290: 20159–20166. 10.1074/jbc.M115.654483 26124270 PMC4536426

[18] Blixt O, Bueti D, Burford B, Allen D, Julien S, Hollingsworth M, et al Autoantibodies to aberrantly glycosylated MUC1 in early stage breast cancer are associated with a better prognosis. Breast Cancer Res. 2011;13: R25 10.1186/bcr2841 21385452 PMC3219186

[19] Taylor-Papadimitriou J, Burchell J, Miles D. W, Dalziel M. MUC1 and cancer. Biochim Biophys Acta—Mol Basis Dis. Elsevier; 1999;1455: 301–313. 10.1016/S0925-4439(99)00055-1 10571020

[20] Seko A, Ohkura T, Ideo H, Yamashita K. Novel O-linked glycans containing 6′-sulfo-Gal/GalNAc of MUC1 secreted from human breast cancer YMB-S cells: Possible carbohydrate epitopes of KL-6(MUC1) monoclonal antibody. Glycobiology. 2012;22: 181–195. 10.1093/glycob/cwr118 21880669

[21] Wu C, Guo X, Wang W, Wang Y, Shan Y, Zhang B, et al N-Acetylgalactosaminyltransferase-14 as a potential biomarker for breast cancer by immunohistochemistry. BMC Cancer. 2010;10: 0–7. 10.1186/1471-2407-10-123 20356418 PMC2873381

[22] Ideo H, Hinoda Y, Sakai K, Hoshi I, Yamamoto S, Oka M, et al Expression of mucin 1 possessing a 3′-sulfated core1 in recurrent and metastatic breast cancer. Int J Cancer. 2015;137: 1652–1660. 10.1002/ijc.29520 25787775

[23] Saeland E, Belo AI, Mongera S, Van Die I, Meijer GA, Van Kooyk Y. Differential glycosylation of MUC1 and CEACAM5 between normal mucosa and tumour tissue of colon cancer patients. Int J Cancer. 2012;131: 117–128. 10.1002/ijc.26354 21823122

[24] Taketa K, Endo Y, Sekiya C, Tanikawa K, Koji T, Taga H, et al A collaborative study for the evaluation of lectin-reactive alpha-fetoproteins in early detection of hepatocellular carcinoma. Cancer Res. 1993;53: 5419–23. Available: http://www.ncbi.nlm.nih.gov/pubmed/7693340 7693340

[25] Gidwani K, Huhtinen K, Kekki H, Van Vliet S, Hynninen J, Koivuviita N, et al A nanoparticle-lectin immunoassay improves discrimination of serum CA125 from malignant and benign sources. Clin Chem. 2016;62: 1390–1400. 10.1373/clinchem.2016.257691 27540033

[26] Tiainen L, Tanner M, Lahdenperä O, Vihinen P, Jukkola A, Karihtala P, et al Bevacizumab Combined with Docetaxel or Paclitaxel as First-line Treatment of HER2-negative Metastatic Breast Cancer. Anticancer Res. International Institute of Anticancer Research; 2016;36: 6431–6438. 10.21873/anticanres.11241 27919965

[27] Eisenhauer EA, Therasse P, Bogaerts J, Schwartz LH, Sargent D, Ford R, et al New response evaluation criteria in solid tumours: Revised RECIST guideline (version 1.1). Eur J Cancer. Pergamon; 2009;45: 228–247. 10.1016/j.ejca.2008.10.026 19097774

[28] Holdenrieder S, Pagliaro L, Morgenstern D, Dayyani F. Clinically meaningful use of blood tumor markers in oncology. Biomed Res Int. Hindawi Limited; 2016;2016: 9795269 10.1155/2016/9795269 28042579 PMC5155072

[29] Soukka T, Härmä H, Paukkunen J, Lövgren T. Utilization of kinetically enhanced monovalent binding affinity by immunoassays based on multivalent nanoparticle-antibody bioconjugates. Anal Chem. 2001;73: 2254–2260. 10.1021/ac001287l 11393849

[30] Kekki H, Peltola M, van Vliet S, Bangma C, van Kooyk Y, Pettersson K. Improved cancer specificity in PSA assay using Aleuria aurantia lectin coated Eu-nanoparticles for detection. Clin Biochem. 2017;50: 54–61. 10.1016/j.clinbiochem.2016.06.015 27818346

[31] Eriksson S, Halenius H, Pulkki K, Hellman J, Pettersson K. Negative interference in cardiac troponin I immunoassays by circulating troponin autoantibodies. Clin Chem. 2005;51: 839–847. 10.1373/clinchem.2004.040063 15718489

[32] R Core Team. R: A Language and Environment for Statistical Computing [Internet]. Vienna, Austria: R Foundation for Statistical Computing; 2018 Available: https://www.r-project.org/

[33] Robin X, Turck N, Hainard A, Tiberti N, Lisacek F, Sanchez JC, et al pROC: An open-source package for R and S+ to analyze and compare ROC curves. BMC Bioinformatics. 2011;12: 77 10.1186/1471-2105-12-77 21414208 PMC3068975

[34] Gion M, Mione R, Leon AE, Dittadi R. Comparison of the diagnostic accuracy of CA27.29 and CA15.3 in primary breast cancer. Clin Chem. 1999;45: 630–637. Available: http://www.ncbi.nlm.nih.gov/pubmed/10222349 10222349

[35] Adamczyk B, Tharmalingam T, Rudd PM. Glycans as cancer biomarkers. Biochim Biophys Acta—Gen Subj. Elsevier; 2012;1820: 1347–1353. 10.1016/j.bbagen.2011.12.001 22178561

[36] Hadjialirezaei S, Picco G, Beatson R, Burchell J, Stokke BT, Sletmoen M. Interactions between the breast cancerassociated MUC1 mucins and C-type lectin characterized by optical tweezers. Kellermayer MS, editor. PLoS One. 2017;12: e0175323 10.1371/journal.pone.0175323 28414807 PMC5393574

[37] Nollau P, Wolters-Eisfeld G, Mortezai N, Kurze AK, Klampe B, Debus A, et al Protein Domain Histochemistry (PDH): Binding of the Carbohydrate Recognition Domain (CRD) of Recombinant Human Glycoreceptor CLEC10A (CD301) to Formalin-Fixed, Paraffin-Embedded Breast Cancer Tissues. J Histochem Cytochem. 2013;61: 199–205. 10.1369/0022155412474823 23275449 PMC3636699

[38] Kubota Y, Fujioka K, Takekawa M. WGA-based lectin affinity gel electrophoresis: A novel method for the detection of O-GlcNAc-modified proteins. PLoS One. Public Library of Science; 2017;12: e0180714 10.1371/journal.pone.0180714 28686627 PMC5501588

[39] Beatson R, Maurstad G, Picco G, Arulappu A, Coleman J, Wandell HH, et al The breast cancer-associated glycoforms of MUC1, MUC1-Tn and sialyl-Tn, are expressed in COSMC wild-type cells and bind the C-type lectin MGL. PLoS One. Public Library of Science; 2015;10: e0125994 10.1371/journal.pone.0125994 25951175 PMC4423978

[40] Chandrasekaran E V., Xue J, Xia J, Khaja SD, Piskorz CF, Locke RD, et al Novel interactions of complex carbohydrates with peanut (PNA), Ricinus communis (RCA-I), Sambucus nigra (SNA-I) and wheat germ (WGA) agglutinins as revealed by the binding specificities of these lectins towards mucin core-2 O-linked and N-linked glycans a. Glycoconj J. 2016;33: 819–836. 10.1007/s10719-016-9678-y 27318477

[41] Bird-Lieberman EL, Neves AA, Lao-Sirieix P, O’Donovan M, Novelli M, Lovat LB, et al Molecular imaging using fluorescent lectins permits rapid endoscopic identification of dysplasia in Barrett’s esophagus. Nat Med. 2012;18: 315–321. 10.1038/nm.2616 22245781

[42] Van Poznak C, Somerfield MR, Bast RC, Cristofanilli M, Goetz MP, Gonzalez-Angulo AM, et al Use of biomarkers to guide decisions on systemic therapy for women with metastatic breast cancer: American Society of Clinical Oncology clinical practice guideline. J Clin Oncol. American Society of Clinical Oncology; 2015;33: 2695–2704. 10.1200/JCO.2015.61.1459 26195705 PMC5478102

