# Supplementary material for: Lectin nanoparticle assays for detecting breast cancer-associated glycovariants of cancer antigen 15-3 (CA15-3) in human plasma
Source: PLoS One. 2019 Jul 25;14(7):e0219480. doi: 10.1371/journal.pone.0219480 (PMC6658058; doi:10.1371/journal.pone.0219480)
Supplement: S1 Fig — Calibration curves of (A) CA15-3 WGA and (B) CA15-3-MGL NPs-lectin assay. Both are linear in range of 1 to 125 U/ml with excellent analytical sensitivity. (PPTX) [file pone.0219480.s001.pptx]

## Slide 1
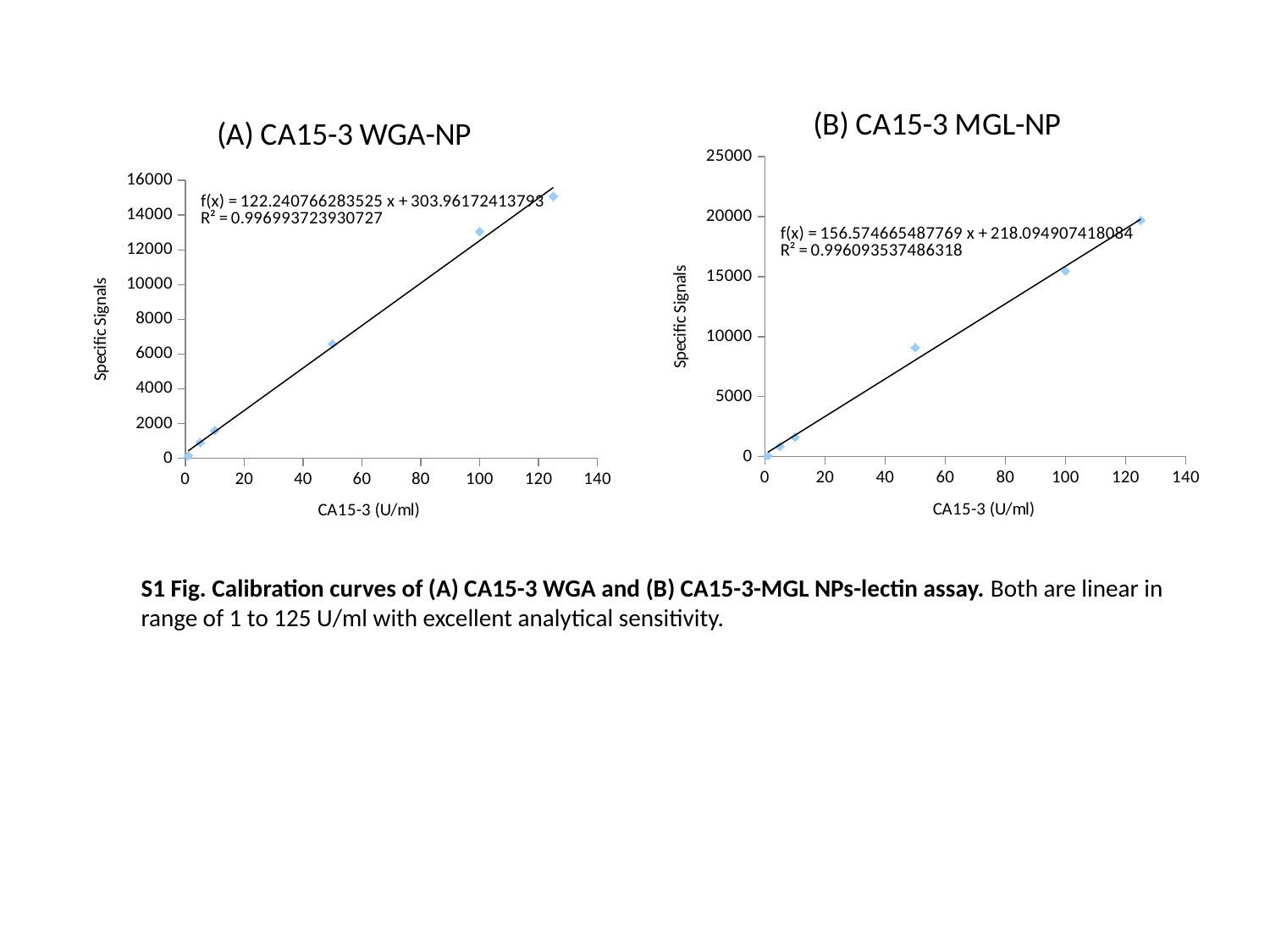

### Chart: (B) CA15-3 MGL-NP
| Category | CA15-3 MGL-NP in TSA-BSA |
|---|---|
### Chart: (A) CA15-3 WGA-NP
| Category | WGA-NP TSA-BSA |
|---|---|S1 Fig. Calibration curves of (A) CA15-3 WGA and (B) CA15-3-MGL NPs-lectin assay. Both are linear in range of 1 to 125 U/ml with excellent analytical sensitivity.
