# Supplementary material for: Lectin nanoparticle assays for detecting breast cancer-associated glycovariants of cancer antigen 15-3 (CA15-3) in human plasma
Source: PLoS One. 2019 Jul 25;14(7):e0219480. doi: 10.1371/journal.pone.0219480 (PMC6658058; doi:10.1371/journal.pone.0219480)
Supplement: S2 Fig — No cross reactivity with CA15-3 lectin assay was observed with ovarian cancer cell line associated CA125 and prostate cancer associated LnCAp PSA (PPTX) [file pone.0219480.s002.pptx]

## Slide 1
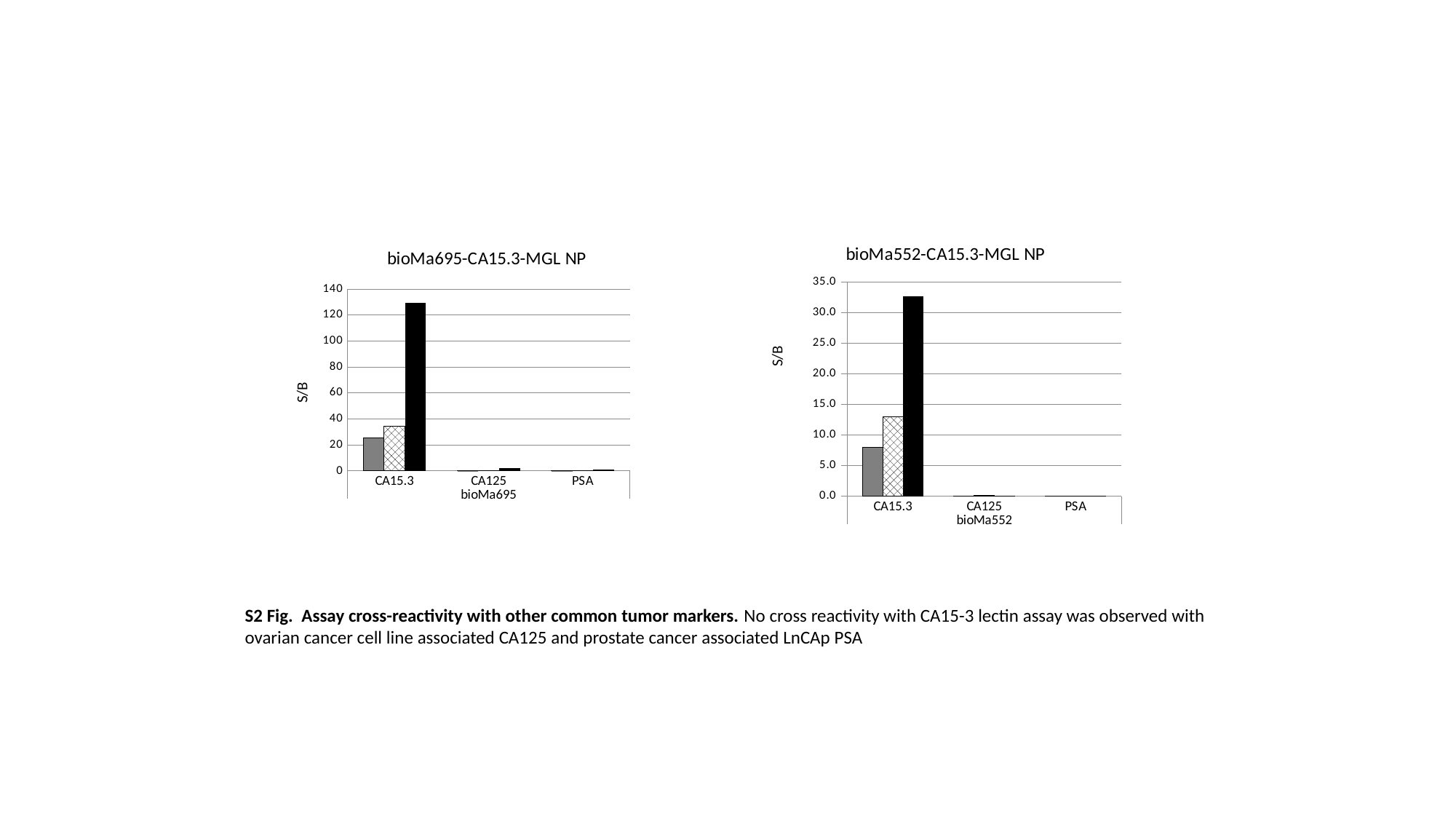

### Chart: bioMa695-CA15.3-MGL NP
| Category | | | |
|---|---|---|---|
| CA15.3 | 25.57608695652174 | 34.59507246376812 | 129.2431884057971 |
| CA125 | 0.12383252818035426 | 0.1389694041867955 | 2.107085346215781 |
| PSA | 0.08166761850371217 | 0.12121644774414621 | 1.1793260993717876 |
### Chart: bioMa552-CA15.3-MGL NP
| Category | | | |
|---|---|---|---|
| CA15.3 | 8.069284937851505 | 13.064700600217401 | 32.681695732312484 |
| CA125 | 0.10095497953615282 | 0.11894295386791977 | 0.12935172553180738 |
| PSA | 0.09606569900687549 | 0.08642680373120122 | 0.14496478202931662 |S2 Fig. Assay cross-reactivity with other common tumor markers. No cross reactivity with CA15-3 lectin assay was observed with
ovarian cancer cell line associated CA125 and prostate cancer associated LnCAp PSA
