# Supplementary material for: Lectin nanoparticle assays for detecting breast cancer-associated glycovariants of cancer antigen 15-3 (CA15-3) in human plasma
Source: PLoS One. 2019 Jul 25;14(7):e0219480. doi: 10.1371/journal.pone.0219480 (PMC6658058; doi:10.1371/journal.pone.0219480)
Supplement: S1 Dataset — Table A. Lectins used. Table B. Concentrations measured from controls and metastatic cases (baseline samples). (DOCX) [file pone.0219480.s003.docx]

**Supplementary dataset**

**Table A. Lectins used.**

| **S.No** | **Lectin** | **Full name** | **Major Carbohydrate binding specificities** |
| --- | --- | --- | --- |
| 1 | SBA | Soybean agglutinin | Terminal α-or β linked GalNAc |
| 2 | SNA | *Sambucus nigra* agglutinin | sialic acid α (2-6) Gal |
| 3 | PNA | Peanut aggluttin | Galβ1-3 GalNAc (terminal) |
| 4 | MAA II | *Maackia amurensis* agglutinin II | α2-3-linked sialic acids |
| 5 | AAL | *Aleuria aurantia* lectin | α1-6Fuc |
| 6 | UEA | *Ulex europaeus* agglutinin | Fucα1-2Gal |
| 7 | PHA-E | *Phaseolus vulgaris* agglutinin-erythroagglutinin | bisecting GlcNAc |
| 8 | RCA | *Ricinus communis* agglutinin | Gal-β1-4GlcNAc |
| 9 | WGA | Wheat germ agglutinin | Terminal *N*-acetylglucosamine or chitobiose |
| 10 | WFA | *Wisteria floribunda* agglutinin | GalNAcα or β- 3 or 6 position of galactose |
| 11 | PSA | *Pisum sativum* agglutinin | α-Mannose |
| 12 | VVL | *Vicia villosa* lectin | Terminal α-or β-linked GalNAc (Tn antigen) |
| 13 | TJA-II | *Trichosanthes japonica* agglutinin | Fuc α 1-2Gal and β-GalNAc |
| 14 | DSL | *Datura Stramonium* Lectin | (β-1,4) linked N-acetylglucosamine oligomers |
| 15 | HPA | *Helix pomatia* agglutinin | GalNAc (Tn antigen) |
| 16 | MGL | Macrophage galactose-type lectin | Terminal α-or β-linked GalNAc |
| 17 | DC SIGN | Dendritic Cell-Specific Intercellular adhesion molecule-3-Grabbing Non-integrin (DC-SIGN) | Nonsialylated Lewis antigens and high mannose-type structures |
| 18 | MMR | Macrophage mannose receptor | Terminal mannose, fucose or N‑acetylglucosamine |
| 19 | MBL | Mannose binding lectin | fucose, mannose/mannan |
| 20 | Siglec-2 | Sia-recognizing Ig-superfamily lectin 2 | Sia-> α6Gal-> β4GlcNac-> βR |
| 21 | Siglec-3 | Sia-recognizing Ig-superfamily lectin 3 | Sia-> α6Gal-> β4GlcNac-> βR |
| 22 | Siglec-5 | Sia-recognizing Ig-superfamily lectin 5 | Sia-> α3Gal-> β4GlcNac-> βR |
| 23 | Siglec-9 | Sia-recognizing Ig-superfamily lectin 9 | Sia-> α3Gal-> β4GlcNac-> βR, 6-sulfated sLe^x^ |
| 24 | Siglec-10 | Sia-recognizing Ig-superfamily lectin 10 | Sia-> α3Gal-> β4GlcNac-> βR |
| 25 | Siglec-11 | Sia-recognizing Ig-superfamily lectin 11 | Sia-> α8Sia-> α3Gal-> β4GlcNac-> βR |
| 26 | Gal-3 | Galectin-3 | galactomannans, mannan |
| 27 | Gal-4 | Galectin-3 | SO_3_->3Galβ1->3GalNAc pyranoside |
| 28 | E-sel | E-Selectin | sLe^x^, near residues might affect affinity |

**Table B.** **Concentrations measured from healthy controls and metastatic cases (baseline samples).** This data was used to generate the ROC curves.

| group | conv. CA15-3 (U/ml) | CA15-3^WGA^ (U/ml) | CA15-31^MGL^ (U/ml) |
| --- | --- | --- | --- |
| control | 15.8 | 1.6 | 1.2 |
| control | 9.1 | 0.3 | 2 |
| control | 23.2 | 1.5 | 0.5 |
| control | 10.8 | 0.4 | 0.2 |
| control | 7.4 | 0.3 | 0.7 |
| control | 27.8 | 1.2 | 4.3 |
| control | 4.9 | 0.4 | 0.2 |
| control | 23 | 1.9 | 2.1 |
| control | 26 | 2.4 | 2.2 |
| control | 5.7 | 2.9 | 8.4 |
| control | 19.5 | 2.5 | 4.3 |
| control | 33.4 | 3.7 | 2.9 |
| control | 9.4 | 0.3 | 40 |
| control | 20.1 | 4.6 | 2.1 |
| control | 8.5 | 1.6 | 1.9 |
| control | 30.4 | 4.3 | 5.7 |
| control | 8.4 | 1.5 | 0.1 |
| control | 6.2 | 0.6 | 0.1 |
| control | 16.1 | 1.1 | 0 |
| control | 7.4 | 4.5 | 0.1 |
| case | 11.4 | 5.8 | 0.8 |
| case | 81.4 | 22.2 | 39.7 |
| case | 49.9 | 34.1 | 4.5 |
| case | 12.6 | 6.5 | 4 |
| case | 52.8 | 24.3 | 16 |
| case | 46 | 14.2 | 75.6 |
| case | 28.2 | 25.2 | 32.7 |
| case | 19.3 | 6.4 | 1.8 |
| case | 11.3 | 5.7 | 2.2 |
| case | 19.5 | 9.2 | 4.9 |
| case | 123.6 | 21.8 | 368.2 |
| case | 82.6 | 40 | 6.2 |
| case | 57 | 17.3 | 205.5 |
| case | 71.1 | 27.4 | 221.3 |
| case | 8.5 | 0.9 | 0.9 |
| case | 204.5 | 69.9 | 52.3 |
| case | 16.9 | 3.2 | 0.2 |
| case | 59.3 | 13.8 | 7.9 |
| case | 16.8 | 5.5 | 2.7 |
| case | 196.9 | 58 | 124.5 |
| case | 29.4 | 6.1 | 4.8 |
| case | 391.7 | 98.3 | 66 |
| case | 17.1 | 4.9 | 9.2 |
| case | 399.5 | 268.8 | 6.8 |
| case | 480.6 | 90.3 | 350.6 |
| case | 11.5 | 3.2 | 2.6 |
| case | 71.2 | 12.7 | 37.1 |
| case | 62.8 | 33.3 | 12.2 |
| case | 13.9 | 5.3 | 14.8 |
| case | 572.9 | 65 | 476.2 |
| case | 144.7 | 28.4 | 659.5 |
| case | 23.7 | 6.5 | 3.6 |
| case | 1165.5 | 180.7 | 383.1 |
| case | 227.9 | 33.6 | 268 |
| case | 10847.2 | 423.6 | 47.6 |
| case | 19.1 | 3.3 | 3.9 |
| case | 35.1 | 3.2 | 35 |
| case | 213.8 | 41.7 | 32.1 |
| case | 12.7 | 5.8 | 2.1 |
| case | 99.9 | 21.7 | 7 |
| case | 18.9 | 2 | 4.4 |
| case | 36.8 | 2.9 | 14.9 |
| case | 15 | 1.5 | 14.4 |
| case | 57.5 | 5.6 | 15.9 |
| case | 47.4 | 10.5 | 7.9 |
| case | 34 | 5.8 | 13.7 |
| case | 2444.4 | 236.4 | 6.8 |
| case | 3 | 2.5 | 0.3 |
| case | 85.9 | 11.9 | 46 |
| case | 63.2 | 16 | 12.3 |
| case | 651.7 | 89.8 | 157.6 |
| case | 7.6 | 3.9 | 1.4 |
| case | 33.1 | 6.6 | 8.1 |
